# Correction: DCID-mediated esterification of carboxylic acids with alcohols under mild conditions

**DOI:** 10.1039/d4ra90051k

**Published:** 2024-05-10

**Authors:** Farzaneh Nasiri, Javad Mokhtari, Salman Taheri, Zohreh Mirjafary

**Affiliations:** a Department of Chemistry, Science and Research Branch, Islamic Azad University PO Box 14515/775 Tehran Iran j.mokhtari@srbiau.ac.ir; b Chemistry & Chemical Engineering Research Center of Iran PO Box 14335-186 Tehran Iran

## Abstract

Correction for ‘DCID-mediated esterification of carboxylic acids with alcohols under mild conditions’ by Farzaneh Nasiri *et al.*, *RSC Adv.*, 2023, **13**, 27385–27390, https://doi.org/10.1039/d3ra04048h.

The authors regret that there were inconsistencies in the mass spectra data in the original electronic supplementary information (ESI).

The authors have repeated their results and provided a new ESI file with new mass spectra data which has been assessed by an independent expert.

The melting point of compound **5c** in Table 2 was incorrect. The correct melting point for compound **5c** is 40–44 °C (ref. 1). The reference below is ref. 17 in the original paper.

The Royal Society of Chemistry apologises for these errors and any consequent inconvenience to authors and readers.

## Supplementary Material
